# Postnatal Mental Health, Hand Washing Practices, and Infant Illness in Rural China

**DOI:** 10.3389/fgwh.2021.735264

**Published:** 2021-11-18

**Authors:** Qi Jiang, Nourya Cohen, Mika Ohtori, Jie Gao, Qingzhi Wang, Evelyn Zhang, Sabrina Zhu, Hannah Johnstone, Yian Guo, Sarah-Eve Dill, Huan Zhou, Scott Rozelle

**Affiliations:** ^1^Freeman Spogli Institute for International Studies, Stanford University, Stanford, CA, United States; ^2^School of Public Health, University of California, Berkeley, Berkeley, CA, United States; ^3^Department of Health Behavior and Social Medicine, West China School of Public Health and West China Fourth Hospital, Sichuan University, Chengdu, China

**Keywords:** postnatal mental health, depression, anxiety, stress, hygiene practices, handwashing practices, infant illness, rural China

## Abstract

**Background:** Maternal mental health problems play an important role in infant well-being. Although western countries have extensively studied the associations between maternal mental disorders, hygiene practices and infant health, little is known in developing settings. This study investigates the correlations between postnatal mental health problems, hand washing practices and infant illness in rural western China.

**Methods:** A total of 720 mothers of infants aged 0–6 months from four poor counties in rural western China were included in the survey. Mental health symptoms were assessed using the Depression, Anxiety, and Stress Scale-21 (DASS-21). Questions about infant illness and hand washing practices followed evaluative surveys from prior studies. Adjusted ordinary least squares regressions were used to examine correlations between postnatal mental health (depression, anxiety, and stress) symptoms, hand washing practices, and infant illness outcomes.

**Results:** Maternal depression, anxiety and stress symptoms were significantly associated with reduced hand washing overall and less frequent hand washing after cleaning the infant's bottom. Mental health symptoms were also associated with a higher probability of infants showing two or more illness symptoms and visiting a doctor for illness symptoms. Individual hand washing practices were not significantly associated with infant illness; however, a composite measure of hand washing practices was significantly associated with reduced probability of infant illness.

**Conclusion:** Postnatal mental health problems are prevalent in rural China and significantly associated with infant illness. Policy makers and practitioners should investigate possible interventions to improve maternal and infant well-being.

## Background

Studies have found that a child's health in the earliest years of life is essential for lifelong health and development (1). However, as many as 5.2 million children under 5 years die from preventable illnesses each year, the majority of whom reside in low- and middle-income countries (LMICs), including China (2). In LMICs, the chance of child and infant illness is much higher due to risk

factors such poor access to clean water and fewer cleaning agents, and the rate of recovery from preventable illness is much lower due to fewer resources and a lack of adequate healthcare (2). Similarly, in rural China, as many as 22% of all infant deaths were found to be associated with diarrheal illness and as many as 71% of rural infant deaths were associated with acute respiratory infections in 2015 (3). Such high rates of fatality due to preventable infant illness requires further study and intervention.

The international literature has established that mother and caregiver hygiene practices are a key factor in child and infant health outcomes in LMICs. The World Health Organization (WHO) has predicted that better hygiene practices could improve over half of diarrheal illnesses worldwide (4). International studies similarly show that poor hygiene practices, particularly among caregivers, are associated with infant and child illnesses (5). A study in Ethiopia found that children under 5 whose mothers had poor hand washing practices were more likely to be admitted to the hospital for episodes of diarrhea (6). Conversely, a study in South Africa found that infants whose caregivers frequently engaged in hand washing practices had reduced incidences of diarrhea and pneumonia (7).

Beyond the relationship between caregiver hygiene practices and child health, postnatal mental health has been linked to infant health outcomes in LMICs. Because infants are most dependent on mothers for care (8), postnatal mental health issues (which may manifest in poor hygiene practices) have been associated with higher rates of infections, diarrheal diseases and hospital visits among infants (9–13). In addition, there is evidence that postnatal mental health issues are associated with higher rates of admission to neonatal care units (14, 15).

While there is not definitive evidence of a causal relationship between postnatal mental health and the hygiene practices of mothers, studies conducted in LMICs have found mental health issues to be correlated to reduced hygiene practices. Two studies of adolescents in LMICs found that those with depressive symptoms had significantly poorer hygiene practices than their peers, including less frequent hand washing and less intention to use soap (16, 17). However, to our knowledge, only one study has examined the association between postnatal mental health and hygiene practices. This study, conducted in Ethiopia, Bangladesh, and Vietnam, found that postnatal mental health issues were significantly associated with poor hygiene practices in the household (18). Hygiene was not a significant focus of the paper, however, and the hygiene measures presented in the study were vague and limited in scope. Therefore, more thorough research in developing settings, is necessary to confirm the correlations between postnatal mental health and hygiene practices.

Like many LMICs, research has found high rates of infant illnesses and poor postnatal mental health in rural areas of China, thus making it an ideal site to examine postnatal mental health and hygiene practices. A study of children under five in rural China found that 48% had experienced diarrhea, 50% had experienced fevers and 60% had spells of coughing in the 2-week period before the survey (19). These rates are comparable to, and in some cases even higher than, other developing countries, where rates of illness in any given 2-week period tend to be around 40–50% for diarrhea (20, 21), 10–50% for fever, and 10–20% for coughing spells (22, 23). Research on postnatal mental health in China has also found rates of depression around 20–25% (24–26), higher than both the 13% global rate of maternal mental disorders but similar to the rate of mental disorders among women in other LMICs (20%) (27).

In this study, the research team aims to investigate the association between postnatal mental health problems, hand washing practices, and infant illness in rural China. More specifically, the study seeks to achieve four objectives. First, we describe the prevalence of postnatal mental health problems, maternal hand washing practices, and infant illness in rural western China. Second, we investigate the correlations between postnatal mental health problems and infant illness. Third, we examine the correlations between postnatal mental health problems and hand washing practices. Finally, we analyze the associations between hand washing practices and child health outcomes. We hypothesize that postnatal mental health problems will be negatively associated with hand washing practices. We also hypothesize that mental health problems will be positively associated infant illness, whereas hand washing will be negatively associated with infant illness.

## Materials and Methods

The data come from the baseline survey of a planned interventional study (not reported in this paper) conducted among low-income families residing in rural areas of Nanchong prefecture in Sichuan Province. According to the National Bureau of Statistics of China, 48% of Sichuan's population are rural residents (28). The average per capita disposable annual income in rural areas of Sichuan is RMB 13,331 ($1906), far lower than the national average of RMB 28,228 ($4,033) (29). These factors allow us to define our study sample as being rural, low-income participants.

This study received ethical approval from the Stanford University Institutional Review Board (Protocol # 44312). All participants provided informed consent to participate in this study. All methods were performed in accordance with the relevant guidelines and regulations.

### Sampling

The research team followed a multi-stage random cluster sampling method. First, within Nanchong prefecture, four nationally designated “poverty counties” were randomly selected. Next, sample townships were selected from within each county. The research team excluded all non-rural townships and rural townships with populations <10,000. Of the remaining townships, 20 townships per county were randomly selected to reach a total of 80 townships. Third, the research team obtained a list of eligible households (households with pregnant women beyond the second trimester or new mothers with infants under 6 months) in each township from the local county-level Maternal and Child Hospital. The research team aimed to recruit 25 such households from each township. In townships with more than 25 eligible households, 25 households were randomly selected to be enrolled in the sample. In townships with fewer than 25 eligible households, all eligible households were included in the study sample. The vast majority of townships had fewer than 25 eligible households, however, with an average of about 13 eligible households per township across the study area. In total, 1,223 eligible participants (pregnant women and new mothers) completed survey interviews. Because this study aims to identify the role of the mother's postnatal mental health in hand washing practices and infant health outcomes, we excluded 353 households whose respondents were pregnant women and 41 households whose respondents were not the mothers of the infants. After dropping 109 mothers with missing key data, our final analytical sample totalled 720 new mothers in 80 rural townships.

### Data Collection

The research team surveyed sample households from November to December 2019. One-on-one survey interviews were conducted with each participant by survey enumerators who were trained to implement the survey instruments for each of the study's main components. The survey collected four blocks of data: postnatal mental health (depression, anxiety, and stress symptoms), hand washing practices, infant illness outcomes and demographic variables.

#### Postnatal Mental Health

To measure the mental health of sampled mothers, mothers were administered the Depression, Anxiety, and Stress Scale-21 (DASS-21). DASS-21 is a 21-item, short-form version of the DASS-42 created by Lovibond and Lovibond (30) and validated in China (31, 32). Participants were given 21 statements about their emotions in the past week, with seven items corresponding to depression, seven to anxiety and seven to stress. Participants were then asked to rank each statement on a scale from 0 to 3 (where 0 = did not apply to me at all and 3 = applied to me most of the time). Scores for depression, anxiety and stress were then calculated by totalling the item scores for each subscale and multiplying by two. Hence, the scores for each subscale could range from 0 to 42. Following to the DASS manual, individuals are considered symptomatic of a given mental health issue if they scored above 9 for depression, 7 for anxiety, and 14 for stress. It is important to note, however, that the DASS-21 only evaluates the symptoms of mental illness and cannot be used for clinical diagnosis.

#### Infant Illness Outcomes

Infant illness outcomes were assessed using questions derived from China Health and Nutrition Survey and the International Food Policy Research Institute (IFPRI) Alive & Thrive baseline survey (33, 34). Mothers were asked to report whether their infant had exhibited specific symptoms in the 2 weeks prior to the survey (including the day of the survey). Symptoms included fever, cough, runny nose, blood in stool, diarrhea, vomiting, difficulty breathing, skin rash, scrapes, bruising, burning, lethargy, ear or eye irritation, and persistent crying. If the mother answered “yes” to a specific symptom, enumerators then asked the mother whether the infant had visited a doctor or for the symptom. For analysis, two dummy variables were created representing infant illness symptoms (1 = infant exhibited 2 or more symptoms in the past 2 weeks; 0 = otherwise) and doctor visits (1 = infant saw doctor for symptoms in the past 2 weeks; 0 = otherwise).

#### Hand Washing Practices

To assess hand washing practices, enumerators asked caregivers questions related to hand washing based on measures used in other studies of hygiene practices (16, 17, 35). In this study, participants were asked to recall the times when they had washed their hands in the previous day (e.g., before or after going to the bathroom, before or after cooking, before or after handling the infant). Enumerators then counted the number of times participants recalled washing their hands in the previous day to create an overall hand washing count for each mother. Additionally, mothers were asked how often (always, often, rarely, or never) they washed their hands when feeding the baby and after cleaning their infant's bottom. The research team then converted the responses into binary variables, where responses of “often” or “always” were considered “frequent practice” and any other answers were considered “infrequent practice.” Enumerators also collected observational data on whether a cleansing agent (such as soap or hand sanitizer) was available at the place of hand washing.

#### Demographic Characteristics

The final survey block collected data on infant and household demographic characteristics. Infant characteristics were obtained from each infant's birth certificate and included the infant's gender, age in months, premature birth status, whether the infant had a low birth weight and whether the infant had siblings. Family characteristics included mother's age, whether the infant's mother and father had graduated from high school, and the value of family assets. To measure the value of family assets, a standardized family asset index was created for the sample using polychoric principal component analysis based on whether each household owned or had access to tap water, a toilet, a water heater, a washing machine, a computer, Internet, a refrigerator, an air conditioner, a motorcycle or electric bicycle, and a car. The family asset index was internally standardized, with higher asset index scores values indicating a higher value of assets relative to other households in the sample (36).

### Statistical Analysis

Our statistical analysis is composed of four parts. First, the research team calculated the overall prevalence of maternal depression, anxiety, and stress symptoms, as well as the overall prevalence of infant illness outcomes and maternal hand washing practices. Second, an adjusted ordinary least squares (OLS) regression was used to identify the correlations between mental health problems and hygiene practices. Hygiene practices include the overall hand washing count, whether the mother frequently washed her hands before feeding the infant, whether mother frequently washed her hands before cleaning the infant's bottom, and whether the household has a cleaning agent at the place of hand washing. Postnatal mental health is a dummy variable for whether the mother of infant shows symptoms of a given mental health issue (depression, anxiety, or stress). In the third and fourth parts of our analysis, adjusted OLS regressions were used to examine the associations between mental health issues and infant illness, and between hygiene practices and infant illness, respectively. In these regressions, the dependent variable is infant illness outcomes, including whether infant has shown at least two symptoms of illness in the past 2 weeks, or whether infant has been taken to the doctor for said symptoms in the past 2 weeks. Variables for postnatal mental health and hygiene practices are the same described above.

All regressions control for potential confounders including infant gender and age, whether the infant was born prematurely, whether the infant had siblings, whether the infant had low birth weight, maternal age, maternal and paternal and education levels, and family asset index score. *P*-values below 0.05 were considered statistically significant. Analyses were conducted in STATA 15.0.

## Results

Table 1 reports the demographic characteristics of our sample. Among the 720 sample infants, 393 infants (55.0%) were male, and the average age of the sample was 2.7 months at the time of the survey. Only 27 infants (3.8%) were born prematurely, and 22 (3.1%) were born with low birth weight. The majority of sample infants were only children, with about 203 infants (28.2%) having siblings. The average age of mothers was about 28 years. Only 283 mothers (39.3%), and 325 fathers (45.1%) had graduated from high school.

**Table 1:** Descriptive statistics of demographic characteristics (*N* = 720).

	***N*/Mean**	**%/SD**
Male infant	393	54.58
Infant age (months)	2.72	2.03
Premature birth	27	3.75
Low birth weight	22	3.06
Infant has siblings	203	28.19
Maternal age (years)	28.02	4.95
Mother graduated high school	283	39.31
Father graduated high school	325	45.14
Family assets (PCA index score)	−0.02	0.99

*Data are presented in mean and standard deviation (SD) for continuous variables and in N and percentage for binary variables*.

Table 2 presents descriptive statistics for maternal mental health symptoms, hygiene practices and infant illness outcomes. Mental health symptoms are presented in Panel A. Within our sample, 93 mothers (12.9%) scored above the cut-off, indicating symptoms of depression. There were 110 mothers (15.3%) who scored above the cut-off for symptoms of anxiety, and 68 mothers (9.4%) who scored above the cut-off for symptoms of stress. Panel B presents the hand washing practices of mothers. The data show that, on average, mothers washed their hands about 6.2 times in the past day. Additionally, 571 mothers (79.3%) stated that they frequently washed their hands before feeding their infants, and 663 mothers (92.1%) reported that they frequently washed their hands after cleaning their infant's bottom. Enumerator observations also show that 565 households (78.5%) had some type of cleaning agent at the place of hand washing. Panel C presents infant health outcomes. The results show that 282 infants (39.1%) showed symptoms of illness at least twice in the last 2 weeks. The most common symptoms include runny nose (31.33%), cough (25.97%), skin rash or dermatitis (21.75%), diarrhea (16.40%), vomiting (11.20%). Among infants with symptoms of illness, 217 infants (30.1%) had been taken to a doctor for their symptoms.

**Table 2:** Descriptive statistics of maternal mental health symptoms, hand washing practices and infant illness outcomes (*N* = 720).

	***N*/Mean**	**%/SD**
**Panel A. Mental health problems**
Mother has symptoms of depression	93	12.9
Mother has symptoms of anxiety	110	15.3
Mother has symptoms of stress	68	9.4
**Panel B. Hand washing practices**
Hand washing count during the previous day	6.2	3.5
Frequent hand washing before feeding the infant	571	79.3
Frequent hand washing after cleaning the infant's bottom	663	92.1
Has cleaning agent at the place of hand washing	565	78.5
**Panel C. Infant illness outcomes**
Infant had two or more illness symptoms in the past 2 weeks	282	39.1
Infant visited the doctor for illness symptoms in the past 2 weeks	217	30.1

*Data are presented in mean and standard deviation (SD) for continuous variables and in N and percentage for binary variables*.

Table 3 reports the OLS correlations between postnatal mental health problems and hand washing practices. Depressive symptoms were significantly associated with less frequent hand washing: mothers with symptoms of depression washed their hands 1.582 fewer times than non-symptomatic mothers, on average (*p* < 0.001). They were also 10.3% less likely to wash their hands before feeding their infants (*p* = 0.020); 8.1% less likely to wash their hands after cleaning their infants' bottoms (*p* = 0.006); and 8.6% less likely to have a cleaning agent at the place of hand washing (*p* = 0.030). Similarly, mothers with symptoms of anxiety washed their hands about 0.989 fewer times than non-symptomatic mothers (*p* = 0.006), and they were 6.7% less likely to wash their hands after cleaning their infants' bottoms (*p* = 0.017). Finally, mothers with symptoms of stress washed their hands 1.595 fewer times in the previous day than non-symptomatic mothers (*p* < 0.001) and were 7.8% less likely to wash their hands after cleaning their infants' bottoms (*p* = 0.024).

**Table 3:** Maternal mental health symptoms and hand washing practices (*N* = 720).

	**Hand washing count in previous day**	**Frequently wash hands before feeding infant**	**Frequently wash hand after cleaning infant's bottom**	**Has cleaning agent**
Depression symptoms	−1.582^***^ (0.384)	−0.103* (0.044)	−0.081^**^ (0.030)	−0.086^*^ (0.040)
Anxiety symptoms	−0.980^**^ (0.361)	−0.025 (0.041)	−0.066^*^ (0.028)	−0.020 (0.036)
Stress symptoms	−1.588^***^ (0.443)	−0.071 (0.051)	−0.077^*^ (0.034)	−0.031 (0.045)

*Standard errors in parentheses. All regressions control for infant gender and age, whether the infant was premature, whether the infant had low birth weight, maternal age, maternal and paternal and education level, and family asset index*.

* *p <0.05*,

** *p <0.01*,

*** *p <0.001*.

Table 4 presents the results of the OLS regression analysis of correlations between postnatal mental health problems (depression, anxiety, and stress symptoms) and infant illness outcomes (symptoms of illness and doctor visits). Infants whose mothers show symptoms of depression have an 19.0% greater probability of exhibiting at least two illness symptoms in the past 2 weeks (*p* = 0.004) and a 10.4% greater probability of visiting the doctor for illness symptoms in the past 2 weeks (*p* = 0.041). Similarly, infants whose mothers displayed symptoms of anxiety were 16.5% more likely to show at least two symptoms of illness in the past 2 weeks (*p* = 0.011) and 13.0% more likely to visit a doctor for their symptoms (*p* = 0.047). Infants of mothers with symptoms of stress were also 12.7% more likely to show at least two symptoms of illness in the past 2 weeks (*p* = 0.011). Stress symptoms were not significantly associated with doctor visits.

**Table 4:** Maternal mental health symptoms and infant illness outcomes (*N* = 720).

	**Infant had two or more illness symptoms in the past two weeks**	**Infant visited doctor for illness symptoms in the past two weeks**
Depression symptoms	0.190^***^	0.104^*^
	(0.053)	(0.051)
Anxiety symptoms	0.165^***^	0.130^**^
	(0.049)	(0.047)
Stress symptoms	0.127^*^	0.044
	(0.061)	(0.059)

*Standard errors in parentheses. All regressions control for infant gender and age, whether the infant was premature, whether the infant had low birth weight, maternal age, maternal and paternal and education level, and family asset index*.

* *p <0.05*,

** *p <0.01*,

*** *p <0.001*.

Table 5 reports the OLS regression between hand washing practices and infant health outcomes. The results find negative but insignificant associations between any individual hand washing practice (overall hand washing count, frequently washing hands before feeding, frequently washing hands after cleaning infant's bottom, and having cleaning agent at the place of hand washing) and infant health outcomes. However, infants of mothers who reported frequently washing hands before feeding and after cleaning their infants' bottoms and had a cleaning agent at the place of hand washing showed a significantly lower prevalence of illness symptoms by 7.4% (*p* = 0.045).

**Table 5:** Hand washing practices and infant illness symptoms (*N* = 720).

	**Infant had two or more illness symptoms in past 2 weeks**
Hand washing count in previous day	−0.003
	(0.005)
Frequently wash hands before feeding infant	−0.002
	(0.045)
Frequently wash hand after cleaning infant's bottom	−0.082
	(0.067)
Has cleaning agent at the place of hand washing	−0.088
	(0.059)
Frequently wash hands before feeding infant and after cleaning infant's bottom and has cleaning agent at the place of hand washing	−0.074^*^ (0.037)

*Standard errors in parentheses. All regressions control for infant gender and age, whether the infant was premature, whether the infant had low birth weight, maternal age, maternal and paternal and education level, and family asset index*.

* *p <0.05*.

## Discussion

This study examined the associations between postnatal mental health, hand washing practices, and infant illness outcomes using detailed survey data from 720 new mothers of infants aged 0–6 months. The results found postnatal mental health problems were significantly associated with reduced hand washing practices, including both overall hand washing count and whether mothers washed hands after cleaning their infant bottoms. Depression and anxiety symptoms were also significantly associated with higher probability of infant illness symptoms and doctor visits. Finally, we found inconsistent negative associations between hand washing practices and infant illness outcomes. Collectively, these results suggest that postnatal mental health is significantly related to infant care and infant health outcomes. Our study underscores the importance of implementing effective interventions to improve the health and well-being of both new mothers and their infants.

The results found that more than one third (39%) of infants had at least two different symptoms of illnesses in the last 2 weeks. Correspondingly, the observed hygiene practices in our sample of rural China were generally poorer than those of high-income countries. On average mothers washed their hands 6.2 out of a possible 14 times during the day. In contrast, a study among the general population of Americans found that respondents reported washing their hands an average of 8.6 times (37). The hand washing count among American new mothers is expected to be even higher, since new parents are prone to cleaning and checking the safety of their infants (38). In addition, 20.7% of mothers did not frequently wash their hands before feeding the infant, 7.9% did not frequently wash their hands after cleaning their infant's bottom, and 21.5% did not have any cleaning agent for hand washing. These findings are consistent with a study conducted in rural China that found that only 23.8% of rural adults practice proper hand washing behaviors, in that they frequently handwashed at key times, used soap, and didn't share a towel with family members after hand washing (39).

Overall, we found significant negative associations between symptoms of mental health problems and hand washing practices. Mothers with symptoms of depression, anxiety and stress showed significantly lower hand washing counts in the previous day and were significantly less likely to frequently wash hands after cleaning their infants' bottoms. These findings are consistent with the few studies that exist on mental health and hygiene, which have similarly found that the two are negatively associated (16–18). Given these findings, it is likely that mental health issues are associated with mothers paying less attention to their infant's hygiene—an association which is common with other child care practices, such as infant nutrition (40–42) and immunizations (9).

Additionally, we found strong associations between maternal mental health issues—particularly depression and anxiety symptoms—and infant illness outcomes. Infants of mothers with symptoms of depression, anxiety and stress were more likely to have at least two illness symptoms in the past 2 weeks. We also found that mother showing symptoms of depression, anxiety, and stress were more likely to have taken their infants to the doctor for symptoms of illness. This is consistent with other studies that show an inverse relationship between mental health and general early childhood outcomes, with depressed mothers more likely to have children who are ill, malnourished, and have low birth weight (9–15).

Surprisingly, our analysis of the associations between hand washing practices and infant illness outcomes yielded weak and inconsistent results. The associations of infant illness to overall hand washing count, frequently washing hands before feeding the infant, frequently washing hands after cleaning the infant's bottom and having cleaning agent at the place of hand washing were all insignificant, although the coefficients were generally negative. This contradicts a number of studies from other LMICs that have found hygiene practices such as hand washing are critical for infant health (5–7). However, when we combine hygiene measures (frequently washing hands before feeding the infant, frequently washing hands after cleaning infant's bottom and having cleaning agent at the place of hand washing) into a single dummy variable, we find frequent combined hygiene practices to be significantly associated with reduced illness symptoms. This suggests that mothers must frequently engage in multiple hygiene practices to effectively protect their infants from illness, which may be particularly challenging for mothers struggling with symptoms of postnatal mental health issues.

Our findings show that postnatal mental health in rural China has far reaching implications for infant health, and thus requires more attention from policymakers and researchers. For instance, proactively including mental health care into perinatal care may mitigate the associations found between poor postnatal mental health and hygiene practices (16–18). This type of mental health support is likely to result in positive behavioral outcomes such as increased engagement and greater attentiveness toward infant health (25, 43–47). Additionally, although infants of depressed mothers showed the highest prevalence of illness, however, hygiene practices were relatively poor and illness symptoms relatively prevalent across the entire sample. Interventions focusing on health and hygiene education may be useful in preventing poor infant health outcomes. Emphasis on when and how to properly wash their hands (such as before feeding their infants or after cleaning the bottom of their infants) is likely to result in better hygiene practices across the community and lower rates of infant illness.

This study has many strengths. It is one of the first to examine the correlations between postnatal mental health, hand washing practices, and infant illness outcomes, and the first paper to do so in China. This study also fills a gap in the literature on postnatal mental health and infant care in rural China, by at women with infants aged 0–6 months, as opposed to previous studies which have rarely sampled women with young children.

We also acknowledge several limitations of this study. First and foremost, because our analysis utilizes cross-sectional data, we are unable to draw temporal or causal conclusions regarding the correlations between mental health, hygiene practices and child health outcomes. It is possible that child illnesses deteriorated maternal mental health, as child illnesses can be distressing for mothers (48). In addition, it is possible that other mental health issues may show different relations to hygiene practices and child illness. For example, obsessive-compulsive symptoms may present as frequent hygiene practices in new mothers (38), which could be protective for child health outcomes. Finally, there may also be unobserved endogenous variables underlying the associations identified in this study, such as the breastfeeding status of infant or whether the infant has siblings at home. Future studies should aim to assess a broader number of mental health issues, including postpartum-specific concerns (49), as well as a broader number of health and nutrition outcomes. Future studies should also follow mothers and their infants over time to better understand the temporal and causal relationships between postnatal mental health, hygiene practices and child health over time.

## Conclusion

This study found that postnatal mental health is significantly associated with hygiene practices and infant health outcomes. The results show significant associations between symptoms of mental health problems and reduced hand washing practices, and between mental health symptoms and increased infant illness symptoms. Infants whose mothers engage frequently in multiple hygiene practices, however, are less likely to exhibit illness symptoms. The findings of this study call for greater attention from researchers and policy makers to further investigate the effects of postnatal mental well-being on infant health, as well as develop and assess the effects of interventions to improve the hygiene knowledge and practices of new mothers on infant health and illness outcomes.

## Data Availability Statement

The original contributions presented in the study are included in the article/supplementary material, further inquiries can be directed to the corresponding author/s.

## Ethics Statement

The studies involving human participants were reviewed and approved by Stanford University Institutional Review Board (IRB Protocol #44312). The patients/participants provided their written informed consent to participate in this study.

## Author Contributions

QJ, NC, MO, EZ, SZ, YG, S-ED, and SR contributed to the conceptualization of the study. QJ, QW, EZ, YG, S-ED, and SR contributed to the acquisition, analysis or interpretation of data. QJ, NC, MO, EZ, SZ, and YG drafted the manuscript. QJ, JG, QW, HJ, S-ED, HZ, and SR made substantial revisions to the manuscript. All authors contributed to the article and approved the submitted version.

## Funding

This research was funded by National Natural Science Foundation of China (No. 71874114). Funding sources played no role in the design of the study, data collection, analysis, or interpretation, or in writing the manuscript.

## Conflict of Interest

The authors declare that the research was conducted in the absence of any commercial or financial relationships that could be construed as a potential conflict of interest.

## Publisher's Note

All claims expressed in this article are solely those of the authors and do not necessarily represent those of their affiliated organizations, or those of the publisher, the editors and the reviewers. Any product that may be evaluated in this article, or claim that may be made by its manufacturer, is not guaranteed or endorsed by the publisher.

## Abbreviations

LMICs: Low- and Middle-Income Countries
DASS-21, Depression, Anxiety: and Stress Scale (short form)
IFPRI: International Food Policy Research Institute.
